# Long term cognitive outcomes of early term (37-38 weeks) and late preterm (34-36 weeks) births: A systematic review

**DOI:** 10.12688/wellcomeopenres.12783.1

**Published:** 2017-10-17

**Authors:** Sarah R. Murray, Susan D. Shenkin, Kirsten McIntosh, Jane Lim, Benjamin Grove, Jill P. Pell, Jane E. Norman, Sarah J. Stock

**Affiliations:** 1 Tommy's Centre for Maternal and Fetal Health, MRC Centre for Reproductive Health, University of Edinburgh Queen’s Medical Research Institute, Edinburgh, EH16 4TJ, UK; 2 Geriatric Medicine Unit, Royal Infirmary of Edinburgh and Centre for Cognitive Ageing and Cognitive Epidemiology, University of Edinburgh, Edinburgh, EH16 4TJ, UK; 3 Royal Infirmary of Edinburgh, University of Edinburgh, Edinburgh, EH16 4TJ, UK; 4 Department of Psychology, University of Edinburgh, Edinburgh, EH16 4T, UK; 5 Section of Public Health, University of Glasgow, Glasgow, G12 8RZ, UK

**Keywords:** Labour, Labour induction, Prematurity, Preterm labour

## Abstract

**Background: **There is a paucity of evidence regarding long-term outcomes of late preterm (34-36 weeks) and early term (37-38 weeks) delivery.  The objective of this systematic review was to assess long-term cognitive outcomes of children born at these gestations.

**Methods: **Four electronic databases (Medline, Embase, clinicaltrials.gov and PsycINFO) were searched.  Last search was 5 ^th^ August 2016. ****Studies were included if they reported gestational age, IQ measure and the ages assessed.  The protocol was registered with the International prospective register of systematic reviews (PROSPERO Record CRD42015015472).  Two independent reviewers assessed the studies.  Data were abstracted and critical appraisal performed of eligible papers.

**Results:** Of 11,905 potential articles, seven studies reporting on 41,344 children were included.  For early term births, four studies (n = 35,711) consistently showed an increase in cognitive scores for infants born at full term (39-41 weeks) compared to those born at early term (37-38 weeks) with increases for each week of term (difference between 37 and 40 weeks of around 3 IQ points), despite differences in age of testing and method of IQ/cognitive testing.  Four studies (n = 5644) reporting childhood cognitive outcomes of late preterm births (34 – 36 weeks) also differed in study design (cohort and case control); age of testing; and method of IQ testing, and found no differences in outcomes between late preterm and term births, although risk of bias was high in included studies.

**Conclusion:  **Children born at 39-41 weeks have higher cognitive outcome scores compared to those born at early term (37-38 weeks).  This should be considered when discussing timing of delivery.  For children born late preterm, the data is scarce and when compared to full term (37-42 weeks) did not show any difference in IQ scores.

## Introduction

Globally, preterm birth rates are rising with 10% of neonates born less than 37 weeks gestation ^1^. Late preterm births (34–36 weeks) account for three quarters of all preterm births ^2^. Early term births (37–38 weeks gestation) have also increased and contribute substantially to an overall decrease in gestational age at delivery. In the US, the average gestational age at delivery has decreased from 40 weeks in 1994 to 39 weeks of gestation in 2004 ^3^.

Early term delivery is associated with increased short term adverse physical morbidity, including respiratory distress syndrome, transient tachypnoea of the neonate and ventilator use, as well as an increased risk of infant mortality at 37 weeks compared to full-term delivery ^4– 6^. It is for this reason that both the Royal College of Obstetricians and Gynaecologists UK (RCOG) ^7^ and the American college of Obstetricians and Gynecologists (ACOG) ^8^ endorse the policy of elective birth after 39 weeks in order to reduce the risk of adverse outcome in infants born before full term (39–40 weeks gestation). There is a paucity of evidence regarding the long-term morbidity of this group, in particular the impact on cognitive function. Advanced gestational age is associated with a lower risk of having special educational need at school ^9^. Davis *et al.* ^10^ has also shown that even amongst the weeks of term advanced gestational age is associated with better neurodevelopment as demonstrated by magnetic resonance imaging (MRI). As obstetric efforts worldwide continue to attempt to reduce stillbirth amongst term deliveries, induction of labour at an earlier gestational age is becoming more common, despite the guidance above, and therefore it is imperative to consider the long-term outcomes of deliveries before term to guide clinicians and parents on optimum timing of delivery.

The association between preterm birth and long-term neurological morbidity is better established with the risk increasing with decreasing gestational age, with extremely preterm babies (≤ 26 weeks) having the worst neurological outcomes ^11, 12^. The aetiology of this is hypothesized to be due to the disruption of the pathways of dendritic arborisation, synaptogenesis and the thickening of the developing cortex ^13^. However, there is less evidence regarding long-term cognitive outcomes of late preterm/early term infants, and given they account for the largest proportion of singleton preterm births more research is necessary. A systematic review of 29,375,675 late preterm infants (34–36 weeks) ^14^, found increased risks of cerebral palsy (RR 3.1, 95% CI 2.3-4.2) and lower likelihood of finishing school in the late preterm born infants (RR 0.96, 95% CI 0.95-0.97), but we could find no prior reviews on cognitive outcomes for early term births.

The aims of this systematic review are to describe the objectively measured cognitive outcomes in childhood up to the age of 18 years i) within each gestational week of term (37–42 weeks) and ii) of late-preterm (34–36 weeks) compared to term (37–42 weeks) deliveries. The results are necessary for informed decision making regarding timing of delivery.

## Methods

This systematic review of the literature was conducted according to the STROBE guidelines ^15^ and reported according to the recommendations of the Preferred Reporting Items for Systematic Reviews and Meta-Analyses (PRISMA) guidelines ^16^ (see Supplementary File 1). The study protocol was registered with the University of York Centre for Reviews and Dissemination International prospective register of systemic reviews (PROSPERO Record CRD42015015472). MEDLINE (1946–2016), EMBASE (1947–2016) and PsycINFO (1945–2016) were searched using a search strategy developed and tested in collaboration with a librarian experienced in literature searching ( Supplementary File 2). The searches were supplemented with a manual search through the reference lists of selected primary articles. A forward citation search was performed on all included studies. The first search date was 12 ^th^ January 2015 and the last search was 5th of August 2016. A subsequent search on Clinicaltrials.gov was performed on 2 ^nd^ June 2017.

### Study selection

One reviewer (JL) screened all titles and abstracts and a second reviewer (BG) independently screened through a 10% sample of the 10,882 articles, by reading the title and abstracts of the first 100 articles of every 1000. The search was updated in August 2016, which yielded an additional 1023 titles and abstracts screened independently by two reviewers (SM, KM). After a consensus was reached, the full texts were retrieved and critically appraised by both reviewers independently (SM, KM). We contacted the individual authors of the included studies to obtain the data necessary to complete the results table. Reasons for exclusion were recorded.

Late-preterm birth was defined as a live birth from 34 to 36+6 completed weeks of gestation. The primary outcome was the results of standardised general intelligence quotient (IQ) tests before age 18 rather than specific domains of cognition. General cognitive ability of a physically and neurologically normal, healthy population of individuals was the key outcome measure recorded. Term birth was defined as a live birth from 37 to 42 completed weeks of gestation.

Studies were included if they reported the range of the participants gestational age, assessment of IQ using a validated score; and the age when IQs was assessed. There were no restrictions by study design, language or method of gestational age assessment. Preterm participants were included as long as there was a clear subgroup of gestational age of 34–36 weeks. Excluded studies included those with: unclear method of cognitive testing; if only selected domains of cognition (e.g. verbal intelligence) were tested; if educational outcomes rather than IQ reported; studies involving high-risk or atypical groups as controls (e.g. multiple births, intra-uterine growth restriction, those with bronchopulmonary dysplasia or brain haemorrhage). Full details of inclusion and exclusion criteria are presented in Supplementary File 3.

The quality of studies was assessed based on the representativeness of the general population, the method of measurement of gestational age and the recording of intelligence testing using the Risk of Bias Assessment Tool for nonrandomized studies (RoBANS) tool ^17^) ( Supplementary File 4). Two independent reviewers extracted data from each paper on study location, design, population, IQ score used and the main results.

The studies differed widely in outcome measures of cognition used, and due to the large heterogeneity between the study designs and methods a meta-analysis was not possible.

## Results

**Figure 1.**
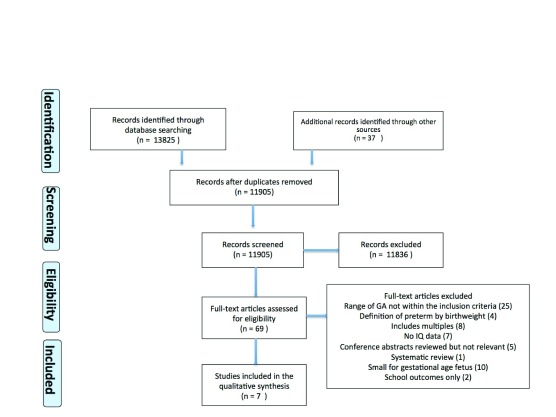
Flowchart showing the study selection process (adapted from the PRISMA flow diagram).

The database and additional record search identified 11,905 articles after removal of duplicates. After exclusions (see flow diagram, Figure 1), six studies and one conference abstract (which reported on both late preterm and outcomes within term and is therefore included in both groups), reporting on 41,344 children/adolescents, were included in the review; four studies comparing the outcomes within term (37–42 weeks) and three studies comparing the outcomes of late preterm delivery (34–36 weeks) with term delivery.

The studies comparing the outcomes within term differed in a number of ways, including: age of testing (1 year, 4 years and 6 years); method of IQ testing (Bayley Scales of Infant Development (BSID), the Stanford-Binet general IQ test and the Wechsler Abbreviated Scale of Intelligence (WASI); details in Supplementary File 5); and the categories of gestational age investigated (37–41 weeks in one study, 37–42 weeks in two studies and 37–43 weeks in one study).

The studies comparing the outcomes of late preterm delivery (34–36 weeks) also differed in a number of ways, including: study design (three prospective cohort studies and one case control study); age of testing (2 years and 13–14 years); and the method of IQ testing (Bayley Scales of Infant Development (BSID) and the Wechsler Intelligence Scale for Children (WISC); details in Supplementary File 5).

Table 1 provides details on the characteristics of the included studies. Three studies ^18– 20^ and one study, which was only available as a conference abstract (on contacting the author no further information was available as it is not yet published) ^21^, reporting on term deliveries (35,711 children/adolescents) and three studies ^22– 24^ (as well as the conference abstract) on late preterm deliveries (5,644 children/adolescents).

**Table 1.** Characteristics of the included studies, ordered by gestational age categories and age of participant testing.

Author, Year (country)	Study design, total n	GA (n)	Control (n)	Age assessed (yrs)	Exclusions	Exposure measurement	Cognitive outcomes *	Adjustment
**Comparison within term (37-42 weeks)**								
Espel *et al*. 2014 ^18^ (USA)	Longitudinal prospective study N = 232	37-41 (232)	-	1	Preterm birth (<37 weeks), Post term birth (>41 weeks)	GA measured from LMP And Ultrasound	BSID II assessed by trained clinicians, no blinding	Maternal ethnicity Marital status Fetal Sex Obstetric complications Maternal education Parity Socio-economic status Birth weight percentile
Rose *et al*. 2013 ^19^ (Chile)	Prospective cohort from an RCT N = 1,562	37-42 (1562)	-	1	Infants born at >42 weeks gestation, birth weight <3kg	GA based on LMP	BSID II performed by trained psychologists, blinding not reported	Fetal sex Socio-economic status Birthweight
Gyamfi Bannerman *et al*. 2014 ^21^ (USA)	Prospective cohort study N = 20,093	37-42 (18,242)		4	Parents with mental retardation or alcohol or drug use	Not reported	Full-scale IQ scores from the Stanford-Binet IQ	Maternal age Maternal education Socio-economic status Smoking
Yang *et al*. 2009 ^20^ (Belarus)	Prospective cohort from an RCT N = 13,824	37 (469) 38 (2100) 42 (171) 43 (10)	39-41 (11, 074)	6.5	Birth weight <2500g	GA from hospital records, 94% confirmed by ultrasound	Full-scale IQ from WASI scores performed by trained pediatricians	Maternal age Marital status Maternal education Parity Smoking Maternal height Parental occupation
**Late preterm vs** **term** **(34 – 36+6)**								
Nepomnyaschy *et al.* 2011 ^22^ (USA)	Prospective cohort study N = 5450	34-36 (400)	37-41 (5050)	2	Mothers <15 yrs, congenital anomalies, multiple births	GA measured by maternal estimated LMP	General, BSID II (converted to a percentile) mental ability, Language by trained interviewer blinded to GA	Maternal ethnicity Maternal age Marital status Fetal sex Obstetric complications Maternal Education Parity Socio-economic status Smoking
Romeo *et al*. 2015 ^23^ (Italy)	Prospective cohort study N -= 119	34-36 (71)	37-41 (48)	2	Abnormal cranial Ultrasound, congenital malformations, SGA	Not reported	MDI of BSID II at 2 yrs Blinding and interviewer training not reported	Fetal sex
Narberhaus *et al*. 2007 ^24^ (Spain)	Case Control study N = 64	34-36 (11)	37-41 (53)	13-14	Mental or physical disability, perinatal complications/hypoxic risk	Not reported	General WISC full scale assessed by the same psychologist	Matched by: Maternal ethnicity Maternal age Fetal sex Maternal education Socio-economic status

*Cognitive Outcomes (details in Supplementary File 4): BSID-II = Bayley Scales of Infant Development (2 ^nd^ Edition); MDI = Mental Developmental Index, PDI = Psychomotor Developmental Index, WISC = Wechsler Intelligence Scale for Children, WASI = Wechsler Abbreviated Scale of Intelligence

Table 2 shows the cognitive outcomes of each week of gestation among term births (range 37–43 weeks). In general, although the studies differed in age at assessment and the IQ test used, all four studies(18–21) showed an increase in cognitive outcome scores for Infants born at full term (39–41 weeks) compared to those children born at early term (37–38 weeks), with statistically significant increases for each week of term. Three of the studies were classed at moderate risk of bias ^18– 20^ and one was at high risk because it did not have any clear information on how gestational age was measured ^21^. Yang *et al.* ^20^ was the only study to measure outcomes up to post-term (43 weeks gestation) and found an inverse U-shaped relationship of IQ score and gestational age. In this large study (n = 13,824), with a moderate risk of bias, full-term (39–41 weeks) was used as a reference group with mean differences in IQ scores reported at early term (37–38 weeks) weeks that were lower than full term and also post term (42–43 weeks), which had a higher mean difference from full term than early term (for full risk of bias see Supplementary File 4). The effect size cannot be summarised due to differences between the studies, but the IQ difference between 37 and 40 weeks was approximately 3 IQ points. This may not be clinically significant at an individual level, but would have an impact at a population level.

**Table 2.** Results of individual studies comparing cognitive outcomes of children born within term (37-42 weeks gestation), ordered by participant age at cognitive testing.

Study (n)	Cognitive test and overall scores	Age at testing (years)	Main findings			Effect size (95%CI)/significance level		Risk of bias ^a^
				Scores				
Espel *et al.* 2014 ^18^ (n = 232)	BSID-II (Bayley scores of infant development) Range 50-150 MDI and PDI ^€^	1	GA ^b^ 37-38 39-40 41	MDI mean ± SD ^c^ 92±NA ^e^ 95±NA 96±NA	PDI mean±SD ^d^ 92.5±NA 97±NA 106±NA	Gestational age as a continuous variable Adj MDI ^f^ b = 2.0 (0.45-3.51), p <0.05 Adj PDI ^g^ b = 3.9 (1.52 - 6.05), p <0.01		Moderate
Rose *et al.* 2013 ^19^ (n = 1562)	Original BSID (Bayley scores of infant development) MDI and PDI	1	GA (n) 37 (45) 38 (260) 39 (469) 40 (604) 41 (184)	MDI mean ± SD 102.6±11.4 103.4±12.3 103.3±12.9 105.1±11.5 105.4±12.2	PDI mean (SD) 94.4±14.9 94.4±14.6 96.6±15.4 98.5±15.1 97.6±14.8	Gestational age as a continuous variable Adj MDI ^h^ b = 0.8 (0.2-1.4), p = 0.025 Adj PDI ^i^ b = 1.4 (0.6-2.1), p = 0.036		Moderate
Gyamfi-Bannerman *et al*. 2014 ^21^ (n = 20,093)	Stanford-Binet IQ Full scale intelligence Quotient (IQ) Range 40-160	4	GA (n) 37 (1290) 38 (2361) 39 (4040) 40 (4816) 41 (3782) 42 (1953)	Mean IQ Scores (95%CI) 95.9 (93.6 - 95.3) 97.6 (95.2 - 96.5) 98.6 (97.1 - 98.1) 99.8 (98.1 - 99.0) 99.8 (99.3 - 100.4) 98.1 (99.0 - 100.5)		Test of linear trend P <0.001		High
Yang *et al.* 2009 ^20^ (n = 13,824)	WASI (Wechsler Abbreviated Scale of Intelligence) Full scale intelligence quotient (IQ) Range 40-160	6.5	GA (n) 37 (469 38 (2100) 39 (11074) 42 (171) 43 (10)		IQ scores NA NA NA NA NA	Unadj mean diff (95%CI) -2.6 (-3.7 - -1.4) 0.6 (-1.1- -0.01) REF ^k^ -1.4 (-3.5 - 0.6) -5.8 (-14 - -2.5)	Adj ^j^ mean diff (95%CI) -1.7 (-2.7 - -0.7) -0.4 (-1.1 - 0.2) REF -0.4 (-2.5 - 1.7) -5.9 (-15 - 3.3)	Moderate

*^b^GA = Gestational age* ^c^MDI = Mental Developmental Index, *SD = standard deviation* ^d^PDI = Psychomotor Developmental Index *^e^NA = not available* *^f,g ^β **c**oefficients represent the actual change in score with each additional week of term, Adj = adjusted for factors ethnicity, parity, maternal age, obstetric risk, birth weight percentile* *^h,i^Means difference coefficients represent the actual change in score with each additional week of term, Adj = adjusted for factors fetal sex, birth weight centile, socio-economic status* *^k^REF = Reference category* *^j^Adjusted for cluster, birth weight, sex, maternal age, maternal height, parental education, and parental occupation*

Table 3 shows the results of the three studies included reporting childhood cognitive outcomes of late preterm birth (34–36 weeks) compared to term birth (37–42 weeks). The abstract by Gyamfi-Bannerman *et al.* ^21^ did not specifically compare late preterm and term deliveries statistically; however, the results were available for each week of gestation and have therefore been recorded in the table. This was the only study that showed a difference in IQ scores between late preterm (mean IQ 92.5) compared to term born children (mean IQ 98.3); however this was not statistically analysed in the published abstract and standard deviations were not available on contacting the authors. This was a large study (n = 20,093), but was assessed as having a high risk of bias as there was no information on the method of gestational age measurement. The two studies using the Bayley scores of infant development did not show a difference in scores between late preterm and term born infants; however this was only done at age 2 and there was no further follow up of the infants. The study by Romeo *et al.* ^23^ was assessed as having a high risk of bias as there was no mention of how gestational age was measured. The study by Narberhaus *et al.* ^24^ provided the longest follow up of the late preterm-born children, testing IQ using the WISC score (Wechsler Intelligence Scale for Children ^25^) at ages 13–14. No statistically significant difference was found in the IQ scores between late preterm (mean IQ 112.7, SD [standard deviation] 13.8) and term born children (mean IQ 113.6, SD 11.5). However, these results should be interpreted with caution as the risk of bias was high (no way to determine selective outcome reporting, only some mentioning blinding of outcome assessments and no clear indication of how gestational age was calculated).

**Table 3.** Results of individual studies comparing cognitive outcomes of children born late preterm (34-36 weeks gestation) to term-born infants (37-41 weeks gestation), ordered by participant age at cognitive testing.

Study (n)	Cognitive test and overall scores	Age at testing (years)	Main findings Late preterm Term Mean±SD Mean±SD	Difference in means (95%CI)/ significance level	Risk of bias
Nepomnyaschy *et al.* 2011 ^22^ n = 5,450 (400 late preterm, 5050 term)	BSID-II (Bayley scores of infant development) Bayley short form – mental ability (MDI) Bayley short form –psychomotor ability (PDI) Scores standardised as short form only used (unadjusted range 50-150)	2	MDI 48.9±10 50.3±10 (Range 92.3 – 174.14) PDI 50.2±9.9 49.9±10 (Range 56.43 – 108.53)	Unadj MDI -1.43 (-2.70 - -0.16), p <0.05 Adj MDI-0.35 (-1.52 - 0.83), p >0.10 Unadj PDI -0.38 (-1.61 - 0.85), p >0.10 Adj PDI -0.33 (-1.58 - 0.91), p >0.10	Moderate
Romeo *et al*. 2015 ^23^ n = 119 (71 late preterm, 48 term)	BSID-II (Bayley scores of infant development) MDI only (range 50-150)	2	96.7±9.3 97.1±6.5	p > 0.05	High
Gyamfi-Bannerman *et al*. 2014 ^21^ n = 20,093 (1,951 late preterm, 18,242 term)	Stanford-Binet IQ+ Full scale intelligence Quotient (IQ) Range 40-160	4	92.5±NA 98.3±NA	NA	High
Naberhaus *et al*. 2007 ^24^ n = 64 (11 late preterm, 53 term)	WISC (Wechsler Intelligence Scale for Children) Full scale intelligence quotient Range 40-160	13-14	112.7±13.8 113.6±11.5	p > 0.05	High

^1^ *Risk of bias is a summary using the RoBANs tool, full details in Supplementary File 4* ^2^MDI = Mental Developmental Index, PDI = Psychomotor Developmental Index ^3^ *Coefficient represent the actual change in score associated with being late-preterm versus full term and Adj. = adjusted for race/ethnicity, age, education, marital birth, father co-residence, household residence, household below poverty* ^4^ *Coefficient represents the actual change in score associated with being late-preterm versus full term and Adj. = adjusted for race/ethnicity, age, education, marital birth, father co-residence, household residence, household below poverty* ^5^ *NA = not available*

## Discussion

### Main findings

In this systematic review of seven studies (reporting on 41,433 children), the four studies investigating IQ scores within term deliveries found that children born at early term (37–38 weeks) had lower IQ scores at ages one, four and six compared to those born at full term (39–41 weeks). One study (n = 13,824) ^20^ found a decrease in IQ score at >42 weeks. In the four studies comparing late-preterm deliveries (34–36 weeks gestation) to their term counterparts there were no differences in cognitive outcomes at ages two, four and 14. Studies were heterogeneous and several were at high risk of bias, and therefore summary effect sizes cannot be reported. No studies were identified comparing outcomes between the ages of four and 14. However, it is useful to consider individual study effect sizes and the potential effect on clinical practice. For example, a three point difference in the Stanford-Binet IQ test between children born at 37 weeks and those born at 41 weeks ^21^ may not be important at an individual level, but this can have important implications at a population level and should be considered along with other factors (estimated birthweight, obstetric risk factors) when clinicians are discussing timing of delivery with parents.

### Strengths and limitations

The strengths of this review include the comprehensive and extensive search strategy, with no language restriction, combined with a detailed pre-defined eligibility criteria for study selection. At the screening stage, to reduce reporter bias, two reviewers independently screened a selected sample to check for accuracy and agreement regarding inclusion of studies. Two reviewers critically appraised all included studies independently. A wide range of cognitive assessments was used in the included studies providing a good overview of the various tests available, but this does make comparison between studies more difficult.

Despite the comprehensive nature of the search, the possibility of missing relevant papers cannot be excluded. We did not have the resources to translate the papers in foreign languages; however we did non-expertly translate to see if any papers fitted the inclusion criteria and none were thought to be relevant. Another limitation was the problems encountered with categorisations of gestational age. A number of studies (22 studies reporting cognitive outcomes of 3,357 infants) only listed <37 weeks of gestational age (all preterm births), which inevitably included those <34 weeks and therefore the whole study was excluded. This may have potentially exacerbated the risk of publication bias as we excluded these studies. Due to limited resources, attempts to contact the individual authors of these studies to see if data was available for 34–36 weeks of gestation was not performed. At delivery, birthweight and gestational age are highly correlated. There is a small but statistically significant correlation between birthweight and cognition in childhood and adulthood (each 1kg increase is associated with 0.13 standard deviation test score increase) ^26^. Some studies did account for birthweight in analyses, and some did not. This may not be appropriate due to their high correlation, and birth weight may be a mediator of the relationship between gestational age and cognition, rather than a confounder. Future studies should report both birth weight and gestational age as a continuous measure, to allow their relative contributions to be measured. Structural equation modelling or similar techniques could be used to model the potential competing causal pathways. We excluded studies with intra-uterine growth restriction (IUGR) because we wanted to study normal healthy singletons, appropriate for gestational age and IUGR may be associated with adverse cognitive outcomes. This review is based on observational data with high levels of between-study heterogeneity, and therefore statistical analysis of the studies was not possible given that the studies were not directly comparable. Limited conclusions can be made regarding the mechanism of action of gestational age on long-term cognitive outcomes because of the nature of the observational data. There were a number of potential sources of bias across the included studies. Although most studies stated how the participants in the studies were chosen in an attempt to reduce selection bias, it is difficult to determine generalizability of the results outwith the populations that were studied. There was a large variation in the number of confounders ( Table 1) adjusted for the various studies, and many did not account for indication for delivery and some did not account for socio-economic factors (strongly associated with cognitive outcomes); therefore there is a risk of residual confounding among the studies.

### Interpretation

Comparing the outcomes within the weeks of term (37–42 weeks), this review has shown that cognitive scores in childhood differ throughout the weeks of term delivery and are lowest in those individuals born in early term gestation (37–38 weeks) when measured at ages one, four and six. Although this review specifically set out to look at cognitive outcomes, the findings are in line with those studies of school performance of individuals born within the different weeks of term. Two large population based studies ^27, 28^ have recently published school outcomes of individuals born within the weeks of term. Smithers *et al.* ^28^ (n = 12,601) showed that children born at 40–41 weeks gestation had the lowest risk of vulnerability at school aged five compared to those born early term (37–39 weeks) or post term (42–45 weeks). Figlio *et al.* ^27^ (n = 1,536,482) showed that children born late term (41 weeks) performed better in school at the age of five through to 18 compared to those born at full term (39 or 40 weeks). Only one of the studies in the review looked at the effect of post-term delivery (>42 weeks) on cognitive outcomes and found those individuals to have a lower score compared to full term (39–40 weeks). This U-shaped relationship has previously been described in the study by MacKay *et al.* (n = 407,503), which found the lowest risk of special educational need at school in those born at 41 weeks gestation compared to those born <41 weeks and >41 weeks ^9^. We identified a previous systematic review published in 2015 that also found a reduction in long term cognitive outcomes of children born early term compared to those born full term, but we were unable to reconcile data included in this previous review with source data ^29^.

The mechanism of early term (37–38 week) delivery leading to lower cognitive outcome scores compared to full term deliveries (39–41 weeks) is likely to be multifactorial. Vohr *et al.* described how brain weight increases rapidly in the last trimester of pregnancy with brain weight at 38 weeks 90% of the weight at full term, which may account for the increased vulnerability of early term infants at school ^5^. For those born post term (>42 weeks), it is thought the increased vulnerability at school age is due to poorer placental perfusion ^30^.

Only three studies were included in the review comparing the cognitive outcomes of children born late preterm (34–36 weeks) with those born at term (37–41 weeks). There were no (statistically or clinically) significant differences in cognition found between these groups at ages two, four and 13. However, the quality of evidence from these observational studies is poor due to high risk of bias (high chance of residual confounding, no outcome assessor blinding and no way to ascertain if selective outcome reporting took place). We therefore do not make any clinical recommendations relating to the timing of delivery, as these observational data cannot be used for this purpose. The included studies all used term deliveries as the control group, but as this includes early term (which, as described above, have lower cognitive outcomes than 39+ weeks) there is a possibility that differences between <37 weeks and later were masked. The conference abstract, although it did not specifically compare the results for late preterm versus term delivery, if we compared late preterm deliveries (mean IQ 92.5) with only full term deliveries (39–41 weeks, mean IQ 98.3) the difference is large and provides further evidence of a partial dilution of the results in treating term deliveries as a continuum. This was a large study; however the data was taken from an old cohort study performed between 1959 and 1966, and many of the variables, including method of gestational age measurement, were not available. Three out of four of the studies had a high risk of bias, and they all assumed homogeneity between the term cases, which, as shown above, is not the case. Although a previous systematic review has shown a clear increase in physical morbidity associated with late preterm delivery ^14^ (34–36 weeks) compared to 37+ weeks, there remains a paucity of evidence regarding long term cognitive outcomes in this group. Future studies should use a full-term delivery group (39–41 weeks) as the control group and adopt uniform gestational age categorizations, ideally with similar outcome measures, to allow for easy comparison between studies. Individual level data should be made available as soon as possible to allow large scale individual participant meta-analysis.

## Conclusion

Overall, this systematic review has found that children born at full term (39–41 weeks) have the highest cognitive outcome scores compared to those born at early term (37–38 weeks). Given the high prevalence of early term deliveries (the fastest growing proportion of singleton births in the US), small differences at an individual level in cognitive outcomes are likely to have a large impact at a population level. Further research is required to look at the potential reasons for this, and to consider outcomes of late-preterm delivery using a suitable control group of full term (39–41 weeks). The findings from this review have important implications for clinicians and the long-term cognitive outcomes based on gestation at delivery should be discussed with parents regarding optimum timing of delivery.

## Supplementary material

Supplementary File 1: PRISMA checklist.

.

Supplementary File 2: Search strategies.

.

Supplementary File 3: Eligibility criteria for study selection.

.

Supplementary File 4: Study quality assessed using the RoBANS tool.

.

Supplementary File 5: Description of the cognitive tests included in the review.
