# Supplementary material for: Long term cognitive outcomes of early term (37-38 weeks) and late preterm (34-36 weeks) births: A systematic review
Source: Wellcome Open Res. 2017 Oct 17;2:101. [Version 1] doi: 10.12688/wellcomeopenres.12783.1 (PMC5721566; doi:10.12688/wellcomeopenres.12783.1)
Supplement: Supplementary file 2 [file wellcomeopenres-2-13850-s0001.tgz › fda338aa-633c-40fa-bbf8-b11f368c9237.docx]

**Supplementary File 2: Search Strategies**

**Ovid MEDLINE/EMBASE (R) In-Process & Other Non-Indexed Citations (1946 to Present),** Searched on: 5 August 2016

| **Searches** | **Results** |
| --- | --- |
| 1 | gestational age/ or premature birth/ or preterm birth/ or preterm infant/ or premature infant/ |
| 2 | (gestation* adj3 age*).tw. |
| 3 | ((pre-term* adj3 birth*) or (preterm* adj3 birth*) or (pre-term* adj3 born*) or (preterm* adj3 born*)).tw. |
| 4 | ((prematur* adj3 born*) or (prematur* adj3 birth*) or (pre-matur* adj3 born*) or (pre-matur* adj3 birth*)).tw. |
| 5 | ((prematur* adj3 bab*) or (prematur* adj3 infan*) or (pre-matur* adj3 infan*)).tw. |
| 6 | ((preterm* adj3 bab*) or (preterm* adj3 infan*) or (pre-term* adj3 bab*) or (pre-term* adj3 infan*)).tw. |
| 7 | 1 or 2 or 3 or 4 or 5 or 6 |
| 8 | ((moderate* adj5 preterm*) or (moderate* adj5 pre-term*) or (moderate* adj5 prematur*) or (moderate* adj5 birth*) or (moderate* adj5 born*)).tw. |
| 9 | ((late adj5 preterm*) or (late adj5 pre-term*) or (late adj5 prematur*)).tw. |
| 10 | ((near term adj5 birth*) or (near term adj5 born*) or (near term adj5 infan*) or (near term adj5 bab*)).tw. |
| 11 | early term.tw. |
| 12 | 8 or 9 or 10 or 11 |
| 13 | 7 or 12 |
| 14 | (preterm* labo?r or pre-term* labo?r or (prematur* adj2 labo?r) or (pre-matur* adj2 labo?r)).tw. |
| 15 | ((preterm* adj2 deliver*) or (pre-term* adj2 deliver*) or (prematur* adj2 deliver*) or (pre-matur* adj2 deliver*)).tw. |
| 16 | (wom#n or matern* or pregnan*).tw. |
| 17 | (resus* or (infan* adj3 dea*) or (infan* adj3 mort*) or ((neonat* adj3 dea*) or (neonat* adj3 mort*))).tw. |
| 18 | (syndrom* or palsy or congenital* or deform*).tw. |
| 19 | (feed* or nutri*).tw. |
| 20 | (heart* or cardio* or lung* or hypox* or pulmon*).tw. |
| 21 | 14 or 15 or 16 or 17 or 18 or 19 or 20 |
| 22 | 13 not 21 |
| 23 | cognition/ or intelligence/ or educational status/ or child development/ |
| 24 | exp mental process$/ or exp aptitude tests/ or exp neuropsychological tests/ or exp psychometrics/ or exp educational measurement/ |
| 25 | (cogniti* or education* or intelligen* or IQ).tw. |
| 26 | (((aptitude or neuropsycholog*) adj5 test*) or ((aptitude or neuropsycholog*) adj5 assess*)).tw. |
| 27 | ((adolescen* adj10 cogniti*) or (teenage* adj10 cogniti*)).tw. |
| 28 | 23 or 24 or 25 or 26 or 27 |
| 29 | 22 and 28 |
| 30 | (nurs* or (care adj3 quali*) or (care adj3 facilit*) or (hospital* adj facilit*) or (hospital* adj care*)).tw. |
| 31 | 29 not 30 |
| **32** | **remove duplicates from 31** |

**PsycINFO (1806 to Present)**

Searched on: 5 August 2016

| **Searches** | **Results** |
| --- | --- |
| 1 | premature birth/ |
| 2 | (gestation* adj3 age*).tw. |
| 3 | ((pre-term* adj3 birth*) or (preterm* adj3 birth*) or (pre-term* adj3 born*) or (preterm* adj3 born*)).tw. |
| 4 | ((prematur* adj3 born*) or (prematur* adj3 birth*) or (pre-matur* adj3 born*) or (pre-matur* adj3 birth*)).tw. |
| 5 | ((prematur* adj3 bab*) or (prematur* adj3 infan*) or (pre-matur* adj3 infan*)).tw. |
| 6 | ((preterm* adj3 bab*) or (preterm* adj3 infan*) or (pre-term* adj3 bab*) or (pre-term* adj3 infan*)).tw. |
| 7 | 1 or 2 or 3 or 4 or 5 or 6 |
| 8 | ((moderate* adj5 preterm*) or (moderate* adj5 pre-term*) or (moderate* adj5 prematur*) or (moderate* adj5 birth*) or (moderate* adj5 born*)).tw. |
| 9 | ((late adj5 preterm*) or (late adj5 pre-term*) or (late adj5 prematur*)).tw. |
| 10 | ((near term adj5 birth*) or (near term adj5 born*) or (near term adj5 infan*)).tw. |
| 11 | early term.tw. |
| 12 | 8 or 9 or 10 or 11 |
| 13 | 7 or 12 |
| 14 | (preterm* labo?r or pre-term* labo?r or (prematur* adj2 labo?r)).tw. |
| 15 | ((preterm* adj2 deliver*) or (pre-term* adj2 deliver*) or (prematur* adj2 deliver*)).tw. |
| 16 | (wom#n or matern* or pregnan*).tw. |
| 17 | (resus* or (infan* adj3 dea*) or (infan* adj3 mort*) or ((neonat* adj3 dea*) or (neonat* adj3 mort*))).tw. |
| 18 | (syndrom* or palsy or congenital* or deform*).tw. |
| 19 | (feed* or nutri*).tw. |
| 20 | (heart* or cardio* or lung* or hypox* or pulmon*).tw. |
| 21 | 14 or 15 or 16 or 17 or 18 or 19 or 20 |
| 22 | 13 not 21 |
| 23 | cognition/ or intelligence/ |
| 24 | exp psychometrics/ or exp educational measurement/ |
| 25 | (cogniti* or education* or intelligen* or IQ).tw. |
| 26 | (((aptitude or neuropsycholog*) adj5 test*) or ((aptitude or neuropsycholog*) adj5 assess*)).tw. |
| 27 | ((adolescen* adj10 cogniti*) or (teenage* adj10 cogniti*)).tw. |
| 28 | 23 or 24 or 25 or 26 or 27 |
| 29 | 22 and 28 |
| 30 | (nurs* or (care adj3 quali*) or (care adj3 facilit*) or (hospital* adj facilit*) or (hospital* adj care*)).tw. |
| 31 | 29 not 30 |
| 32 | remove duplicates from 31 |
| **33** | **limit 32 to yr="1945-Current"** |
