# Supplementary material for: Long term cognitive outcomes of early term (37-38 weeks) and late preterm (34-36 weeks) births: A systematic review
Source: Wellcome Open Res. 2017 Oct 17;2:101. [Version 1] doi: 10.12688/wellcomeopenres.12783.1 (PMC5721566; doi:10.12688/wellcomeopenres.12783.1)
Supplement: Supplementary file 3 [file wellcomeopenres-2-13850-s0002.tgz › 34dfcaab-620a-49e5-825c-c1064c9cd264.docx]

**Supplementary File 3: Eligibility criteria for study selection**
