# Supplementary material for: Long term cognitive outcomes of early term (37-38 weeks) and late preterm (34-36 weeks) births: A systematic review
Source: Wellcome Open Res. 2017 Oct 17;2:101. [Version 1] doi: 10.12688/wellcomeopenres.12783.1 (PMC5721566; doi:10.12688/wellcomeopenres.12783.1)
Supplement: Supplementary file 4 [file wellcomeopenres-2-13850-s0003.tgz › 4de1d9ce-d462-44b8-9614-bb3db3c8b11e.docx]

**Supplementary File 4: Study quality assessed using the RoBANs* tool**

*RoBANS (Risk of Bias Assessment Tool for Nonrandomised Studies)
