# Supplementary material for: Long term cognitive outcomes of early term (37-38 weeks) and late preterm (34-36 weeks) births: A systematic review
Source: Wellcome Open Res. 2017 Oct 17;2:101. [Version 1] doi: 10.12688/wellcomeopenres.12783.1 (PMC5721566; doi:10.12688/wellcomeopenres.12783.1)
Supplement: Supplementary file 5 [file wellcomeopenres-2-13850-s0004.tgz › ec9dd1f0-053f-436b-a971-8fc0648f32be.docx]

**Supplementary File 5: Description of the cognitive tests included in the review.**

| **Measure** | **Description and Sources** |
| --- | --- |
| Bayley scores of infant development (BSID) | - Original Bayley scores – monitor neurodevelopmental outcomes up to the age of 3. This is split into: - The Mental development index (MDI) which measures distinct cognitive, receptive lauguage and expressive language scales and; - The Psychomotor development index (PDI) which measures fine and gross motor skills - BSID-II is the revised bayley scores which was published in 1993 - The Bayley short form includes a subset of score from BSID-II which includess some assessments from both the MDI and the PDI - Raw scores are converted into standard scores based on the the child’s chronological age (mean 100, SD 15 with a range from 50-150) - Scores are interpreted as below:   ≤ 69 = significantly delayed performance  70-84 = mildly delayed performance  85-114 = within normal limits  ≥ 115 = accelerated performance |
| WISC (Weschler Intelligence Scale for Children)  Full scale intelligence quotient | - Intelligence test for children between the ages of 6 and 16. - A Full scale IQ should be generated representing a child’s general intellectual ability. It provides five primary index scores (verbal comprehension index, visual spatial indexm fluid reasoning index, working memory index, and processing speed index) - Scores follow that of general IQ scores   <70 – Impaired or delayed  70-79 – borderline  80-89 – low average  90-110 – average  110-119- high average  120-129 – above average  >130 – gifted/superior |
| WASI (Wechsler Abbreviated Scale of Intelligence)  Full scale intelligence quotient (IQ) | - IQ test designed to measure intelligence and cognitive ability in adults and older adolescents - Most commonly used intelligence test - Scores follow that of general IQ scores   <70 – Impaired or delayed  70-79 – borderline  80-89 – low average  90-110 – average  110-119- high average  120-129 – above average  >130 – gifted/superior |
| Stanford-Binet IQ  Full scale intelligence quotient (IQ) | - Cognitive ability and intelligence test - Can be used from the age of two - Scores follow that of general IQ scores   <70 – Impaired or delayed  70-79 – borderline  80-89 – low average  90-110 – average  110-119- high average  120-129 – above average  >130 – gifted/superior |
