# Supplementary material for: Long term cognitive outcomes of early term (37-38 weeks) and late preterm (34-36 weeks) births: A systematic review
Source: Wellcome Open Res. 2017 Oct 17;2:101. [Version 1] doi: 10.12688/wellcomeopenres.12783.1 (PMC5721566; doi:10.12688/wellcomeopenres.12783.1)
Supplement: Peer review history

# Peer review: Referee response for version 1

**DOI:** 10.21956/wellcomeopenres.13850.r27478

**Published:** 2017-12-07

**Authors:** Nana Hyldig, Gitte Zachariassen

**Affiliations:** 1 Hans Christian Andersens Hospital, Odense University Hospital, University of Southern Denmark, Odense, Denmark

Thank you for the opportunity to review this very well written manuscript on cognitive outcomes of early term and late preterm births. A few thoughts to consider are listed below:

 Abstract

1. In the result section seven studies are mentioned – as in table 1 in the manuscript. For early term births – four studies are mentioned – as in the manuscript. For late preterm births – four studies are mentioned – but only three studies are mentioned in the manuscript including table 1?
2. Definitions also in abstract: late preterm (34-36 weeks), early term (37-38 weeks), full term (39-41 weeks) and term (37-40 weeks)? In conclusion “full term” are also defined as “37-42 weeks” – confusing! In the manuscript in Methods and study design “37-42 completed weeks” are defined as “term birth”.

 

 Method section

1. The last search was more than one year ago. We recommend that an updated search before publication.
2. In the attached PRISMA statement document the authors report that the summary measure is reported on page 10. This is, however, not identifiable in the manuscript. Please report the summary measure / statistical method used to calculate the effect estimates in the method section.
3. At PROSPERO the authors state that they will assess risk of bias by using GRADE, but in the manuscript they have used RoBANS.
4. It is very impressing that one author have screened 10,882 titles and abstracts. The last part of the first paragraph in “Study selection” it says that a second author screened 10% of the articles. How was consensus reached for the remaining 90% of the articles?

 

 Discussion

1. Exclusion of studies including infants <34 does not induce risk of publication bias as the studies are published.
2. Only one study have adjusted for obstetric complications (caesarean sections?)and no information on the fetus/infants.
  Often late preterm and early term born infants are delivered early due to a sick fetus or a sick mother – both with implication on the fetal brain and post natal development.
3. Why were the infants in the studies delivered late preterm or at early term? How many caesarean sections in these groups compared to full term infants? Maybe these infants have a lower IQ due to other circumstances than being born at early term?
  It is mentioned shortly in discussion section under “the mechanism of early term……” but need further discussion.

 

 Conclusion in both abstract and manuscript:

 …”for discussion with parents regarding optimum timing of delivery.” Do you mean counselling parents who wish or request a caesarean section? Or all parents including those with sick foetuses? Should be specified in the conclusion.

We have read this submission. We believe that we have an appropriate level of expertise to confirm that it is of an acceptable scientific standard, however we have significant reservations, as outlined above.

# Peer review: Referee response for version 1

**DOI:** 10.21956/wellcomeopenres.13850.r27305

**Published:** 2017-11-20

**Authors:** Marjolein Kok

**Affiliations:** 1 Academic Medical Center, Department of Obstetrics and Gynecology, University of Amsterdam, Amsterdam, Netherlands

This is a well written systematic review on long term cognitive outcome of late preterm and early term infants. The strength of this systematic review exposes a weakness at the same time. The detailed pre-defined eligibility criteria of IQ scores limit the number of eligible studies and thus gives an insight in the limitations of current long term follow-up studies. There is no uniformity in report on gestational age, outcome measures and confounding factors, making it difficult to draw conclusions from the aggregated data. The authors are aware of this short-fall and suggest themselves that future studies should use a full-term delivery group (39–41weeks) as the control group and adopt uniform gestational age categorizations, with similar outcome measures, to allow for easy comparison between studies. Although the results of this study contributes to the knowledge on this subject, the impact is limited due to above mentioned limitations and the fact that the difference in IQ is only 3 points. I agree however with the reviewers that this may not be clinically significant at an individual level, but would have an impact at a population level. I have no suggestions for changes.

I have read this submission. I believe that I have an appropriate level of expertise to confirm that it is of an acceptable scientific standard.

# Peer review: Referee response for version 1

**DOI:** 10.21956/wellcomeopenres.13850.r27265

**Published:** 2017-11-20

**Authors:** Jennifer M. Walsh

**Affiliations:** 1 Murdoch Children’s Research Institute, Melbourne, Vic, Australia; 2 Royal Women's Hospital, Melbourne, Vic, Australia; 3 Royal Children's Hospital, Melbourne, Vic, Australia

This systematic review attempts to identify differences in IQ scores in 2 comparison groups. The first group are term infants, by comparing early term 37-38 weeks with full term 39-41 weeks' gestation and by identifying differences with each gestational week. The second group compares late preterm infants' IQ scores, gestation 34-36 weeks, with term infants' IQ scores, gestation 37-42 weeks' gestation.

 There is clearly a paucity of data in this regard and this study highlights this.

 The search criteria were well identified by the researchers. The methods well explained and the results clearly outlined. The aims of the study are clear and very important to publish in order to highlight the importance for clear decisions regarding timing of delivery if that is possible.

 The difficulty I have as a clinician, is in the interpretation of the results and I think needs some further qualification in the discussion by the authors. 

 The study gives us a clear message that term infants are better off being delivered at 39-41 weeks' gestation in order to maximise their IQ potential. However, the biggest difficulty in being able to interpret and extrapolate the results shown, is that we have no idea for the reason for early term birth versus full term birth and if it's different or similar between the groups in these studies. The researchers can only deal with what has been published but have made no comment regarding the indications for delivery and the potential impact that might have on the results if the studies were adjusted for this. Potentially, the infants born 37-38 weeks were electively delivered/induced but more likely is that there were a mix of both clear indications for delivery and elective deliveries and this may have been very different to the 39-41 week indications for delivery and contribute to the differences found.

 Of the 4 articles looking at term infants IQ scores only Espel et al. 2014 (the smallest study) adjusted for obstetric complications. Although clearly there are differences in the IQ scores of the 2 groups and this is important, can this be helped? The only way to truly disentangle this is to look prospectively at term infants delivered electively, which is an almost impossible study to do with the numbers needed to evaluate the outcome measures. Alternatively adjustment for the reason for delivery particularly if there's an indication that can impact on the infant long term should be done.

 The preterm data has smaller numbers, the largest study was adjusted for obstetric complications on analysis but similarly the delivery indications are paramount to teasing out the answer to this question. The researchers have clearly highlighted in the discussion that potentially including infants born 37-38 weeks in the comparison group have diluted the results to show no significant difference. Which in itself is interesting. Is being born at 37 weeks as bad as being born at 34 weeks? The studies are generally small making differences difficult to establish but adding the late preterm data from the Gyamfi-Bannerman et al. 2014 study has shown there are likely differences in IQ to be found.

 A recent study by Cheong et al. ^1^ showed that moderate and late preterm (MLPT) children do worse on all domains of assessment using the BSID II compared with term born controls, though moderate preterm children were included (32-33 weeks'), within the MLPT group, there was little evidence of an association between gestational age at birth and neurodevelopment or social-emotional development at age 2. The group acknowledged that there were potential differences between the MLPT infants' in the study compared with other MLPT infants as they were all born in a tertiary setting and hence may have been sicker. The rates of induction and caesarean section are well delineated in the paper. 

 The question of term IQ differences is a very interesting and worthwhile subject to pursue researching.

I have read this submission. I believe that I have an appropriate level of expertise to confirm that it is of an acceptable scientific standard, however I have significant reservations, as outlined above.

## References

[1] CheongJLDoyleLWBurnettACLeeKJWalshJMPotterCRTreyvaudKThompsonDKOlsenJEAndersonPJSpittleAJ: Association Between Moderate and Late Preterm Birth and Neurodevelopment and Social-Emotional Development at Age 2 Years. *JAMA Pediatr*.2017;171(4) : 10.1001/jamapediatrics.2016.4805 e164805 10.1001/jamapediatrics.2016.4805 28152144
